# *Pelargonium graveolens* Attenuates Rotenone-Induced Parkinson’s Disease in a Rat Model: Role of MAO-B Inhibition and In Silico Study

**DOI:** 10.1007/s12035-025-04727-6

**Published:** 2025-02-08

**Authors:** Rana M. Merghany, Salma A. El-Sawi, Asmaa F. Aboul Naser, Mohamed A. Salem, Shahira M. Ezzat, Sherifa F. A. Moustafa, Meselhy R. Meselhy

**Affiliations:** 1 Department of Pharmacognosy, National Research Centre, 33 El Buhouth St, Cairo, 12622 Egypt; 2 Department of Therapeutic Chemistry, National Research Centre, 33 El Buhouth St, Cairo, 12622 Egypt; 3 Department of Pharmacognosy and Natural Products, Faculty of Pharmacy, Menoufia University, Gamal Abd El Nasr St., Shibin El Kom, 32511 Menoufia Egypt; 4 Department of Pharmacognosy, Faculty of Pharmacy, Cairo University, Kasr El-Aini Street, Cairo, 11562 Egypt; 5 Department of Pharmacognosy, Faculty of Pharmacy, October University for Modern Sciences and Arts (MSA), Giza, 12451 Egypt

**Keywords:** Parkinson’s disease, α-Synuclein, Neuro-inflammation, Oxidative stress, MAO-B, *Pelargonium graveolens*

## Abstract

Parkinson’s disease (PD), the second most common neurodegenerative condition, is primarily characterized by motor dysfunctions due to dopaminergic neuronal loss in the Substantia Nigra (SN), with oxidative stress playing a significant role in its progression. This study investigates the neuroprotective potential of *Pelargonium graveolens* (Thunb.) L’Hér leaves in a rotenone-induced PD rat model. The total ethanolic extract and its fractions, obtained via Diaion HP-20 column chromatography, were evaluated for monoamine oxidase-B (MAO-B) inhibition *in vitro*. The 50% methanol fraction (PG50) demonstrated the highest MAO-B inhibition (IC_50_ 5.26 ± 0.12 µg/ml) compared to the reference drug selegiline (IC_50_ 0.021 ± 0.003 µg/ml). In a rotenone-induced PD rat model, PG50 (100 mg/kg, *p.o*.) alleviated motor deficits (assessed via the wire hanging test), and restored norepinephrine, dopamine, and serotonin levels. PG50 and l-dopa reduced α-synuclein levels by 367.60% and 377.48%, respectively. Oxidative balance was restored with increased glutathione (23.12%) and decreased malondialdehyde (164.19%) in brain tissues. PG50 significantly reduced serum TNF-α (572.79%) and IL-6 (70.84%) levels, and improved succinate dehydrogenase (14.47%) and lactate dehydrogenase (7.74%) activities in brain tissues. Histopathological alterations in the SN were also ceased. UPLC-MS/MS analysis identified 61 metabolites, including 32 flavonoids, 13 phenolic acids, 7 coumarins, 5 phenolic glycosides, and 4 dicarboxylic acids, with in silico docking showing strong MAO-B binding by methoxylated flavonoids like methoxyluteolin dimethyl ether (docking score: − 8.0625 kcal/mol), surpassing that of safinamide (− 8.2615 kcal/mol). These findings suggest that *P. graveolens* holds promise as a neuroprotective agent against rotenone-induced PD.

## Introduction

Parkinson’s disease (PD) is considered the second neurodegenerative ailment that affects mainly elderly people (1–2%) worldwide [1]. The principal feature of this disease is motor impairments [2]. This feature attempts to the dopaminergic neuronal loss in the region of substantia nigra (SN) [3]. Also, PD is accompanied by oxidative stress, mitochondrial dysfunction, and inflammation in different brain regions [4]. Further, the pathological feature is mainly characterized by the occurrence of α-synuclein (α-Syn) aggregates (Lewy bodies) [4].

Unfortunately, patients with PD experience progressive disability and reduced quality of life. The cost of illness worsens as PD progresses, placing an economic burden on the healthcare system, society, production rate, and patients themselves [5]. Synthetic dopamine (DA) precursors (e.g., levodopa), DA agonists (e.g., pergolide), and monoamine oxidase-B (MAO-B) inhibitors (e.g., selegiline) are frequently used to manage PD, but they can lead to serious side effects such as cardiopathy and dyskinesia. Furthermore, extended use of these drugs can result in a gradual decrease in their effectiveness [6–9]. As a result, there is a growing need for safe and affordable therapies from natural products.

The genus *Pelargonium* belongs to the family Geraniaceae, which embraces about 830 species that have been widely used by traditional healers in Africa as calming agents since ancient time [10]. *Pelargonium* is characterized by the high content of phenolic compounds and flavonoids [11]. For instance, *Pelargonium* species unveiled the presence of flavonols like myricetin, quercetin, isorhamnetin, and kaempferol, along with their glycosides or methyl ethers as the primary flavonoid components within the *Pelargonium* genus [12]. Furthermore, a variety of species in the *Pelargonium* genus produce significant amounts of both hydrolyzable (ellagitannins) and non-hydrolyzable (proanthocyanidins) tannins [12]. These compounds are recognized for their important role as antioxidants, anti-inflammatory agents, and inhibitors of MAO enzyme [13] and α-Syn aggregation [14, 15]. For instance, aqueous ethanolic extract of *P. sidoides* improved the symptoms of LPS-induced sickness behavior [16]. Furthermore, methanol extracts from the leaves of *P. radens* and *P. zonale* showed cytoprotective and anti-inflammatory properties *in vitro* [17]. Moreover, proanthocyanidins extracted from *P. sidoides* roots exhibited anti-inflammatory properties by preventing the death of fibroblasts induced by LPS, reducing the release of IL-8 and prostaglandin E2 from fibroblasts, as well as IL-6 from leukocytes [18].

High content of phenolic compounds and flavonoids detected in *P. graveolens*, an extensively cultivated *Pelargonium* species in Egypt for its rose-scented geranium oil, can be our guide in this study for assaying its activity against PD [11, 19], where the two classes demonstrate antioxidant and anti-inflammatory activities, and may contribute to MAO enzyme inhibitory [13] and the anti α-Syn protein aggregation activities [14, 15]. Although different studies have focused on the chemical composition and the biological activities of the essential oil of *P. graveolens*, to our knowledge, no in-depth phytochemical and pharmacological studies have been conducted on the non-volatile constituents of this species. However, some studies have pointed to the antioxidant and anti-inflammatory activities of the crude extract of *P. graveolens* [20–23] as well as its anti-acetylcholinesterase inhibition activity [24].

This study aims to investigate the neuroprotective potential of *P. graveolens* fractions against PD. The bioactive fraction was identified based on its inhibitory activity against the MAO-B enzyme *in vitro* and subsequently evaluated for its neuroprotective efficacy in a rotenone-induced PD rat model. To establish a relationship between the observed activity and the chemical composition, comprehensive metabolome profiling of the bioactive fraction was performed using ultra-performance liquid chromatography-tandem mass spectrometry (UPLC-MS/MS). Additionally, an in silico study was conducted to explore the interaction of annotated metabolites with the MAO-B enzyme, correlating their binding affinities with the observed anti-PD effects.

## Materials and Methods

### Plant Material

The leaves of *P. graveolens* were collected in April 2023 from the Experimental Station of Medicinal and Aromatic Plants, Faculty of Pharmacy, Cairo University, Giza, Egypt. The plant material was authenticated by Eng. Therese Labib (A consultant of plant taxonomy, Ministry of Agriculture, Egypt). A voucher specimen (O.S.156) was placed at the herbarium of the National Research Centre, Egypt.

### Preparation of the Total Extract and the Fractions

A sample (1 kg) of the air-dried and powdered leaves was extracted with 80% aqueous ethanol (3 L × 5) by maceration. The combined alcoholic extract was then evaporated under reduced pressure to give 80 g of dry extract (EE). This extract was suspended in water (40 ml), sonicated, and applied to a Diaion-HP20 column (5 cm diameter × 80 cm height). Successive elution was performed using water, 50% aqueous methanol, and finally 100% methanol. Evaporation of the solvents for each fraction yielded a 100% water fraction (12 g), a 50% methanol fraction (50 g), and a 100% methanol fraction (14 g).

### Drugs and Chemicals

Rotenone, selegiline, and vitamin C were purchased from Sigma (St. Louis, MO, USA) and l-dopa was obtained from Merck and Co. Inc. (NJ, USA). All chemicals employed in this study were of the analytical grade.

### *In Vitro* Antioxidant DPPH Scavenging Activity

The DPPH free-radical scavenging activity was evaluated using a spectrophotometer at 517 nm according to Chen et al. (1999) [25]. Serial concentrations of the different samples (10, 50, and 100 μg/mL) were evaluated and vitamin C was used as a reference drug.

% inhibition = (*A0* − *A1* / *A0*) × 100, where *A0* is the absorbance of DPPH^·^ solution (control) and *A1* is the absorbance of the tested samples. Results are expressed as IC_50_.

### *In Vitro* MAO-B Enzyme Inhibitory Activity

The *in vitro* MAO-B enzyme inhibitory activity was determined by the kynuramine deamination assay using a SpectraMax M5 fluorescence plate reader (Molecular Devices, Sunnyvale, CA, USA) with an excitation (320 nm) and emission (380 nm) wavelength according to Gogineni et al. (2017) [26]. A fixed concentration of substrate (kynuramine 50 μM) and a concentration–response curve of inhibitors (0.01 to 100 μg/mL) were used to determine the IC_50_ values. The IC_50_ values were calculated using XLFit software. Selegiline was used as a reference drug.

### *In Vivo* Study of the Anti-Parkinson’s Disease Activity

#### Animals and Ethics

Male Wistar albino rats (160–180 g) were obtained from the Animal House, National Research Centre, Egypt. All animals were retained in regular plastic cages with free access to water and food, maintained under environmentally controlled conditions. Animals were reserved for 2 weeks for adaptation before beginning the experiments. All anesthetic and handling techniques with animals fulfilled the ethical guidelines of the Medical Research Ethics Committee of the National Research Centre in Egypt (approval no: 6667082022).

#### Acute Toxicity Study

The acute toxicity test was conducted in compliance with the Organisation for Economic Co-operation and Development (OECD) guideline 423 [27]. To assess the acute toxicity of the active fraction (PG50), 24 male Wistar albino rats (160–180 g) were divided into four groups (negative control and PG50 at three doses—50, 100, and 200 mg/kg b.wt). Animals were observed for 14 days [28]. No dead rats were detected during the experimental period, signifying that the fraction is safe. Thus, the dose of 100 mg/kg b.wt was selected for the in vivo study.

#### Induction of Parkinsonism

To induce experimental PD in rats, rotenone was combined with a mixture of DMSO and sunflower oil in a ratio of 1:9 v/v. Rats received rotenone injections (1.5 mg/kg, *s.c.*) every 48 h for a period of 12 days [29]. Concurrently, treatment with PG50 (100 mg/kg, *p.o.*) and l-dopa (10 mg/kg, *p.o*.) was administered alongside rotenone injections every 24 h for the same 12-day duration [30].

#### Experimental Design

After a 2-week adaptation period, 30 male Wistar albino rats (weighing 160–180 g) were randomly assigned to five groups (*n* = 6 per group) as outlined as follows:

- Group 1 (normal control)
  - Vehicle: Subcutaneous injections of a vehicle (20 μl DMSO/mL in sunflower oil, 1:9 v/v).
- Group 2 (normal rats + PG50)
  - Control treatment: oral administration of PG50 at a dose of 100 mg/kg.
- Group 3 (PD rats)
  - Induction of PD: subcutaneous injections of rotenone at a dose of 1.5 mg/kg.
- Group 4 (PD rats + PG50)
  - Treatment: rats received rotenone injections (1.5 mg/kg, *s.c.*) along with oral administration of PG50 (100 mg/kg).
- Group 5 (PD rats + l-dopa):
  - Treatment by reference drug: subcutaneous injections of rotenone (1.5 mg/kg) combined with oral treatment of l-dopa at a dose of 10 mg/kg.

The rotenone injections were administered every 48 h for a total of 12 days, while PG50 and l-dopa were administered every 24 h with the rotenone injections for the same duration.

Forty-eight hours after the final treatment, all animals were anesthetized with midazolam (10 mg/kg, *i.p*.) and euthanized by decapitation for subsequent analysis [31].

#### Behavioral Study

Daily observation of the rats was done to follow up on the development of PD symptoms such as bradykinesia and rigidity. Rats were further assessed for their behavior by the wire hanging test, where rats were suspended by their forelimbs on a metal rod (40 cm length and 0.50 cm diameter) positioned 50 cm high above the surface. The time the rat remained on the rod (maximum 60 s) was noted [32].

#### Blood and Tissue Samples

Blood samples were pinched from the retro-orbital plexus of the rats into dry test tubes and centrifuged at 300 × *g* for 15 min. The serum was then separated and kept at − 20 °C to assess the levels of IL-6 and TNF-α [33].

The whole brains were separated directly after decapitation and washed with ice-cold isotonic saline. The washing solution was then removed from the brains using absorbent paper. The brains were weighed, homogenized in 50 mM phosphate buffer (pH 7.4), and centrifuged at 300 × *g* for 10 min at 4 °C. The supernatants were kept at − 20 °C to assess the levels of α-synuclein (α-Syn), norepinephrine (NE), dopamine (DA), serotonin (5-HT), glutathione (GSH), and malondialdehyde (MDA) as well as the activities of superoxide dismutase (SOD), lactate dehydrogenase (LDH), and succinate dehydrogenase (SDH) [33].

#### Estimation of Different Biochemical Markers

The level of α-Syn in brain tissues was quantified using an ELISA kit (Cloud-Clone Company, TX, USA) according to the method of Cerri et al. (2018) [34]. The levels of NE, DA, and 5-HT were determined by the method of Zagrodzka et al. (2000) [35], using high-performance liquid chromatography with electrochemical detection (HPLC-ECD). The levels of GSH and MDA as well as the activity of SOD in brain tissues were determined according to Moron et al. (1979) [36], Wills (1966) [37], and Kono (1978) [38], respectively. The levels of IL-6 and TNF-α in serum were determined by an ELISA kit (Cloud-Clone Company, TX, USA) according to the method of Sun et al. (2012) [39]. The activities of SDH and LDH in brain tissues were estimated according to Shelton et al. (1957) [40] and Babson et al. (1973) [41], respectively.

#### Histopathological Assay

Samples of the brain SN regions were fixed in 10% neutral-buffered formalin, embedded in paraffin wax, cut at 5-μm thickness, and stained with hematoxylin and eosin (H&E) for analysis [42].

### Ultra-Performance Liquid Chromatography

A sample (1 mg) of PG50 was dissolved in 1 mL of aqueous methanol (50% v/v, UPLC-grade) and sonicated for 5 min [11]. The metabolites from this sample were separated using an RP High Strength Silica (HSS) T3 C18 column (100 mm × 2.1 mm with 1.8-μm diameter particles) within a Waters UPLC system (Acquity, Waters Corporation, USA). The injection volume was 2 μL, and the flow rate was set to 0.3 mL/min. Chromatographic conditions included water with 0.1% formic acid as mobile phase A and acetonitrile with 0.1% formic acid as mobile phase B. The separation gradient was designed as follows: 0–1 min at 1% mobile phase B, 1–11 min with a linear increase from 1 to 40% B, 11–13 min with a linear increase from 40 to 70% B, 13–15 min with a linear increase from 70 to 99% B, 15–16 min with 99% B, 16.0–17.0 min with a linear decrease from 99 to 1% B, and 17.0–20.0 min with 1% B. The column temperature was maintained at 40 °C.

### Tandem Mass Spectrometry Acquisition and Annotation of Metabolites

Mass spectra were captured using a high-resolution Orbitrap mass analyzer (Q Exactive system, Thermo Fisher Scientific, Waltham, MA, USA). Data was acquired in data-dependent acquisition (DDA) mode in both positive and negative modes, scanning within the range of 50 to 1500 m/z. Following MS^1^ analysis, the five most intense precursor ions were selected for subsequent dissociation. The ion spray voltage and positive ion voltage were both set at 3500 V, negative ion voltage at 3000 V, utilizing ion source heated electrospray ionization (HESI). The sheath gas pressure was 40 psi; auxiliary heating gas pressure stood at 15 psi; ion source heating temperature was 300 °C; collision energy was cycled between 20, 40, and 60 V; MS^1^ resolution was 70,000; and MS^2^ resolution was 17,500. Metabolite annotation was primarily based on retention times, generated chemical formulas, and fragmentation patterns from Xcalibur 2.1 software, when compared to the different MS databases (METLIN [43], HMDB [44], and MassBank [45]), and reported literature [11].

### Molecular Docking Study

For the assessment of in silico MAO-B enzyme inhibition activity, the study utilized AutoDock (AD) inbuilt vina PyRx 0.8 version. The X-ray crystal structure of the target protein MAO-B was obtained from the Protein Data Bank (PDB ID: 2V5Z), while the 3D structures of the ligands were obtained from the PubChem database.

The protein preparation involved removing water particles, eliminating extra chains, protonation, and energy minimization using the MMFF94 force field. The ligands, including two control drugs [safinamide (co-crystallized ligand) and selegiline] and 61 metabolites, were also energy-minimized.

Validation of the docking protocol was performed by redocking the co-crystalized ligand, and the binding interactions were visualized and analyzed using Discovery Studio 2021. Docking results were evaluated based on the binding energy score (-kcal/mol) and root mean square deviation (rmsd) values.

### Statistical Analysis

Statistical analysis was carried out using one-way analysis of variance (ANOVA) with Costat Software. All data were expressed as mean ± SD (*n* = 6 for the in vivo study and *n* = 3 for the *in vitro* study).

$$\%\mathrm{Change}\;\mathrm{from}\;\mathrm{the}\;\mathrm{Normal}\;\mathrm{control}\;\mathrm{group}=\lbrack(\text{Normal control mean}-\text{Treated mean})/\text{Normal control mean}\rbrack\times100$$

$$\%\mathrm{Change}\;\mathrm{from}\;\mathrm{the}\;\mathrm{PD}\;\mathrm{group}=\lbrack(\text{PD group mean}-\text{Treated mean})/\text{PD group mean}\rbrack\times100$$

$$\%\mathrm{Improvement}=\lbrack\text{Treated mean}-\text{PD mean})/\text{Normal control mean}\rbrack\times100$$

Groups having the same letters are non-significantly different, while those having different letters are significantly different at *p* < 0.05.

## Results

### *In Vitro* Antioxidant DPPH Scavenging Activity of the Total Extract and Its Fractions

**Fig. 1:**
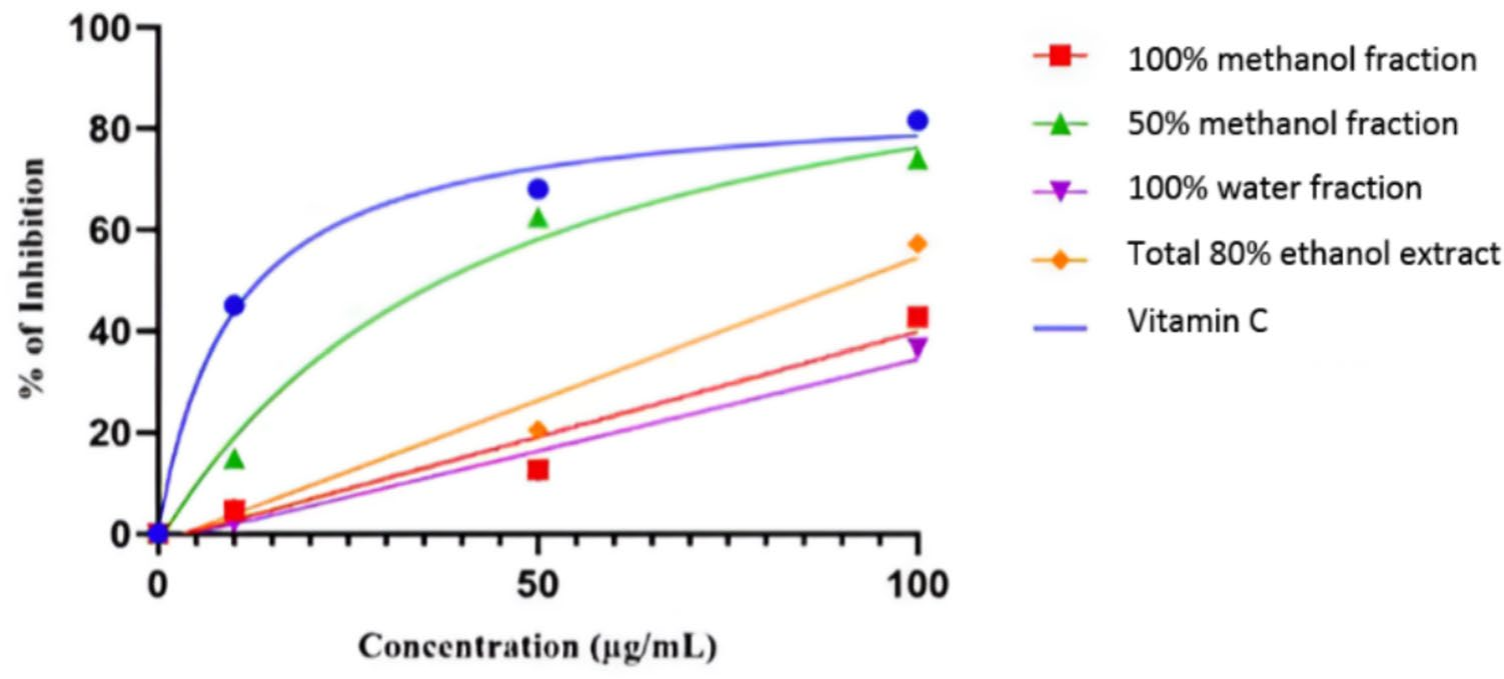
Concentration–response inhibition profile for extract/fractions of *P. graveolens* to evaluate their antioxidant activity *via* DPPH scavenging assay

**Fig. 2:**
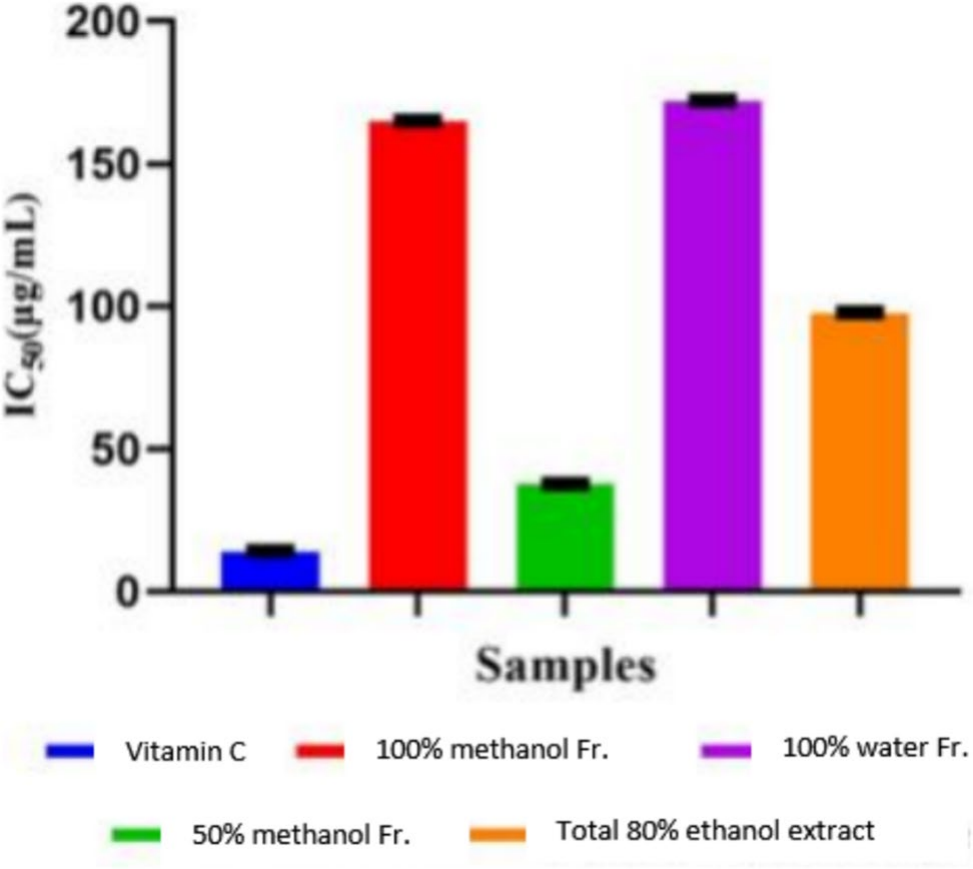
IC_50_ values (µg/ml) of total extract/fractions of *P. graveolens* assessed for antioxidant activity *via* DPPH scavenging assay

The *in vitro* antioxidant activity of the total 80% ethanol extract and its three fractions (100% water fraction, 50% methanol fraction, and 100% methanol fraction) were evaluated using the DPPH scavenging assay. Percent of inhibition was calculated at three different concentrations: 10, 50, and 100 µg/mL (Fig. 1). The results were expressed as IC_50_ values, demonstrating significant differences in the antioxidant activity of the tested extract/fractions and vitamin C (Fig. 2). Vitamin C exhibited a high efficacy as an antioxidant, with an IC_50_ value of 13.97 ± 0.2 µg/ml. Notably, the 50% methanol fraction (PG50) emerged as the most potent among all fractions, displaying an IC_50_ of 37.59 ± 0.20 µg/ml, which is even more effective than the total 80% ethanol extract (IC_50_ of 97.54 ± 0.26 µg/ml). In contrast, the 100% methanol fraction showed a significantly higher IC_50_ value of 164.78 ± 0.27 µg/ml, indicating lower antioxidant activity. The 100% water fraction (IC_50_ of 171.89 ± 0.339 µg/ml) also exhibited the least antioxidant potential among the fractions tested.

### *In Vitro* MAO-B Enzyme Inhibitory Activity of the Total Extract and Its Fractions

The preliminary anti-PD ability of the total 80% ethanol extract and its three fractions (100% water fraction, 50% methanol fraction, and 100% methanol fraction) was evaluated by estimating their inhibitory activity against MAO-B enzyme, an enzyme responsible for the breaking down of different neurotransmitters mainly DA that is important for the coordination of many nerve and muscle cells in the brain. Results showed that the 50% methanol fraction (PG50) showed the highest inhibition of MAO-B enzyme (IC_50_ value of 5.26 ± 0.12 µg/ml), versus the reference drug selegiline (IC_50_ value of 0.021 ± 0.003 µg/ml). Based on the *in vitro* results, PG50 was assessed for its neuroprotective activity in a PD animal model*.*

### *In Vivo* Evaluation of the Anti-Parkinson’s Disease Activity of the Active Fraction

#### Behavioral Study

The ability of PG50 to control the symptoms of PD was evaluated at the end of the experiment by using the wire-hanging test. Results presented in Table 1 indicated no significant difference in the time spent by normal rats receiving PG50 compared to the normal control group. However, a significant reduction of 70.73% was observed in PD rats compared to the normal control group. Treatment with PG50 and l-dopa enhanced the behavioral observations of the rats by 46.34% and 51.70%, respectively, in relation to the PD rats.

**Table 1:** Effect of PG50 on the behavior of PD rats using the wire hanging test/second

Group	Time the rat remained on the rod (s)
Gp-1: Normal control	20.50^a^ ± 2.50
Gp-2: Normal rats + PG50	19.80^a^ ± 2.10 (− 3.41)
Gp-3: PD rats (rotenone-induced)	6.00^c^ ± 1.70 (− 70.73)
Gp-4: PD rats + PG50 (100 mg/kg b.wt./24 h)	15.50^b^ ± 2.30 [+ 158.33]
Gp-5: PD rats + l-dopa (10 mg/kg b.wt./24 h)	16.61^b^ ± 1.50 [+ 176.66]

Data are expressed as mean ± SD of six rats in each group. Groups having the same letters are non-significantly different, while those having different letters are significantly different at *p* < 0.05. Values between brackets represent % of change versus the normal control group. Values between parentheses represent % of change versus the PD group. The “+ or “−” signs indicate increase or decrease in the time, respectively

#### Estimation of Different Biochemical Markers

The level of α-Syn was determined in the brain tissues of different groups as a specific Parkinsonism biomarker (Table 2). Results showed no significant difference in the level of α-Syn after administration of PG50 to normal rats, while a significant increase was observed in PD rats by 453.58%, as compared to the normal control group. Treatment of PD rats with PG50 and l-dopa showed improvement in α-Syn levels by 367.60% and 377.48%, respectively, versus the PD rats.

**Table 2:** Effect of PG50 on the level of α-synuclein and neurotransmitters in PD rats

Group	α-Syn (pg/ml)	DA (μg/g tissue)	NE (μg/g tissue)	5-HT (μg/g tissue)
Gp-1: Normal control	52.94^c^ ± 5.50	5.70^a^ ± 0.21	5.63^a^ ± 0.15	7.23^a^ ± 0 0.83
Gp-2: Normal rats + PG50	55.06^c^ ± 6.40 (+ 4.00)	5.30^a^ ± 0.50 (− 7.01)	4.86^a^ ± 0.15 (− 13.67)	7.10^a^ ± 0.32 (− 1.79)
Gp-3: PD rats (rotenone-induced)	293.07^a^ ± 22.18 (+ 453.58)	2.80^c^ ± 0.26 (− 50.87)	1.96^d^ ± 0.35 (− 65.18)	2.50^a^ ± 0.34 (− 65.42)
Gp-4: PD rats + PG50 (100 mg/kg b.wt./24 h)	98.46^b^ ± 8.50 [− 66.40]	3.80^b^ ± 0.35 [+ 35.71]	3.30^b^ ± 0.32 [+ 68.36]	4.30^b^ ± 0.62 [+ 72.00]
Gp-5: PD rats + l-dopa (10 mg/kg b.wt./24 h)	93.23^b^ ± 7.30 [− 68.18]	4.20^b^ ± 0.25 [+ 50.00]	4.30^b^ ± 0.30 [+ 119.38]	4.53^b^ ± 0.55 [+ 81.20]

Data are expressed as mean ± SD of six rats in each group. Groups having the same letters are non-significantly different, while those having different letters are significantly different at *p* < 0.05. Values between brackets represent % of change versus the normal control group. Values between parentheses represent % of change versus the PD group. The “+” or “−” signs indicate increase or decrease in the level, respectively

Similarly, there was no significant difference in the levels of DA, NE, and 5-HT in brain tissues of normal rats after administration of PG50, as compared to the normal control group. However, PD rats showed a significant decrease in DA, NE, and 5-HT levels by 50.87%, 65.18%, and 65.42%, respectively, with respect to the normal control group. Treatment with PG50 and l-dopa enhanced DA levels by 17.54% and 24.56%, and NE by 23.80% and 41.56%, respectively, compared to those in PD rats. Also, 5-HT levels were enhanced by 24.89% and 28.07%, respectively, in relation to the PD rats (Table 2).

Regarding the oxidative stress markers, normal rats that received PG50 showed no significant difference compared to the normal control group. In contrast, PD rats exhibited a significant decrease in SOD activity and GSH levels by 70.81% and 60.41%, respectively, while MDA level increased significantly by 230.56% compared to the normal control group. However, PD rats treated with PG50 and l-dopa demonstrated a significant increase in SOD activity by 31.40% and 42.96%, respectively, when compared to PD rats. Also, GSH levels improved by 23.12% and 31.54%, respectively, while MDA levels decreased significantly by 164.19% and 181.65%, respectively, in comparison to PD rats (Table 3).

**Table 3:** Effect of PG50 on oxidative stress and mitochondrial dysfunction markers in PD rats

Group	GSH (µg/g tissue)	MDA (µmol/m protein)	SOD (µg/m protein)	SDH (μmol/m protein)	LDH (μmol/m protein)
Gp-1: Normal control	25.77^a^ ± 0.64	2.29^c^ ± 0 0.27	79.32^a^ ± 3.43	3.73^a^ ± 0.21	4.26^a^ ± 0.21
Gp-2: Normal rats + PG50	24.10^a^ ± 0.36 (− 6.48)	2.40^c^ ± 0.26 (+ 4.80)	78.23^a^ ± 4.14 (− 1.37)	3.50^a^ ± 0.40 (− 6.16)	4.13^a^ ± 0.15 (− 3.05)
Gp-3: PD rats (rotenone-induced)	10.20^c^ ± 1.50 (− 60.41)	7.57^a^ ± 0.70 (+ 230.56)	23.15^d^ ± 2.40 (− 70.81)	1.29^c^ ± 0.18 (− 65.41)	1.90^c^ ± 0.10 (− 55.39)
Gp-4: PD rats + PG50 (100 mg/kg b.wt./24 h)	16.16^b^ ± 1.76 [+ 58.43]	3.80^b^ ± 0.30 [− 49.73]	48.06^c^ ± 5.78 [+ 107.60]	1.83^b^ ± 0.30 [+ 41.86]	2.23^b^ ± 0.15 [+ 17.36]
Gp-5: PD rats + l-dopa (10 mg/kg b.wt./24 h)	18.33^b^ ± 0.76 [+ 79.70]	3.40^b^ ± 0.36 [+ − 55.08]	57.23^b^ ± 2.85 [+ 147.21]	2.06^b^ ± 0.15 [+ 59.68]	2.63^b^ ± 0.15 [+ 38.42]

Data are expressed as mean ± SD of six rats in each group. Groups having the same letters are non-significantly different, while those having different letters are significantly different at *p* < 0.05. Values between brackets represent % of change versus the normal control group. Values between parentheses represent % of change versus the PD group. The “+” or “−” signs indicate increase or decrease in the level/activity, respectively

The effect of PG50 on the mitochondrial dysfunction initiated by Parkinsonism was evaluated by measuring the activities of the SDH and LDH enzymes (Table 3). The results revealed no significant reduction in their activities in normal rats who received PG50, in relation to the normal control group. A significant reduction in the activities of SDH and LDH (by 65.41% and 55.39%, respectively), was noted in PD rats, compared to the normal control group. However, treatment with PG50 and l-dopa resulted in an improvement in SDH activity by 14.47% and 20.64%, respectively, compared to the PD rats. Meanwhile, LDH activity was enhanced by 7.74% and 17.13%, respectively, in relation to the PD rats.

Concerning IL-6 and TNF-α levels in serum, no significant difference was observed in the serum of normal rats that received PG50 compared to the normal control group, while the PD rats showed a significant increase in their levels by 143.79% and 812.50%, respectively, as compared to the normal control group. Treatment with PG50 and l-dopa recorded a significant decrease in IL-6 levels by 70.84% and 105.72%, respectively, while TNF-α levels reached 572.79% and 723%, respectively in comparison to the PD rats (Table 4).

**Table 4:** Effect of PG50 on the levels of IL-6 and TNF-α of PD rats

Group	IL-6 (pg/ml)	TNF-α (pg/ml)
Gp-1: Normal control	53.16^c^ ± 5.50	218.86^d^ ± 16.37
Gp-2: Normal rats + PG50	55.96^c^ ± 6.71 (+ 5.26)	228.08^d^ ± 15.32 (+ 4.21)
Gp-3: PD rats (rotenone-induced)	129.60^a^ ± 10.80 (+ 143.79)	1997.10^a^ ± 81.14 (+ 812.50)
Gp-4: PD rats + PG50 (100 mg/kg b.wt./24 h)	91.94^b^ ± 9.83 [− 29.05]	743.48^b^ ± 78.46 [− 62.77]
Gp-5: PD rats + l-dopa (10 mg/kg b.wt./24 h)	73.40^bc^ ± 7.10 [− 43.36]	414.45^c^ ± 31.50 [− 79.24]

Data are expressed as mean ± SD of six rats in each group. Groups having the same letters are non-significantly different, while those having different letters are significantly different at *p* < 0.05. Values between brackets represent % of change versus the normal control group. Values between parentheses represent % of change versus the PD group. The “+” or “−” signs indicate increase or decrease in the level, respectively

#### Histopathological Analysis

**Fig. 3:**
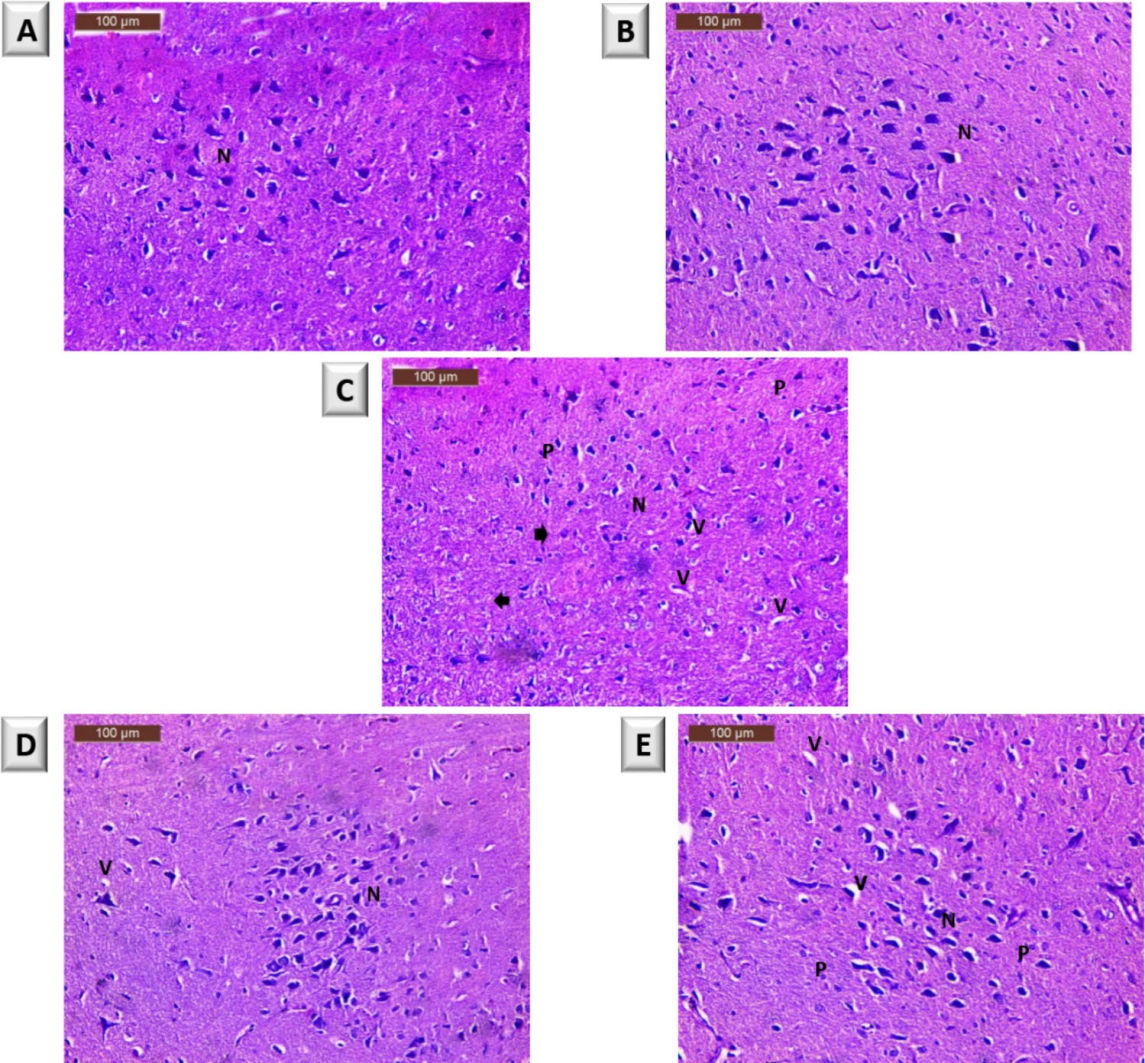
Photomicrographs of substantia nigra of different rat groups. **A** Normal control group. **B** Normal rats + PG50. **C** PD rats. **D** PD rats + PG50. **E** PD rats + l-dopa. N, normal neuron. V, vacuoles. P, pyknotic nuclei. Arrowhead: reduced in number of neurons

Histopathological examination of SN of different rat groups was assessed. In Fig. 3, the normal control group exhibited normal SN neurons with intact nuclei and no signs of degeneration (Fig. 3A). Further, normal rats received PG50 alone appeared nearly similar to the normal control group (Fig. 3B). In contrast, examination of H&E-stained sections from PD rats showed neurodegeneration in the SN (arrowhead), a reduction in number of normal neurons (N), an increase in vacuoles (V), and the presence of pyknotic nuclei (P) (Fig. 3C). PD rats treated with PG50 and l-dopa showed improvements, displaying nearly normal neurons with mild vacuolation and few pyknotic nuclei (Fig. 3D and Fig. 3E, respectively).

### Annotation of Metabolites

Metabolite profiling of the bioactive fraction of *P. graveolens* (PG50) was performed, and 61 compounds were tentatively identified based on comparisons of their retention times and MS data with various MS databases (METLIN [43], HMDB [44], and MassBank [45]) as well as relevant literature.

**Fig. 4:**
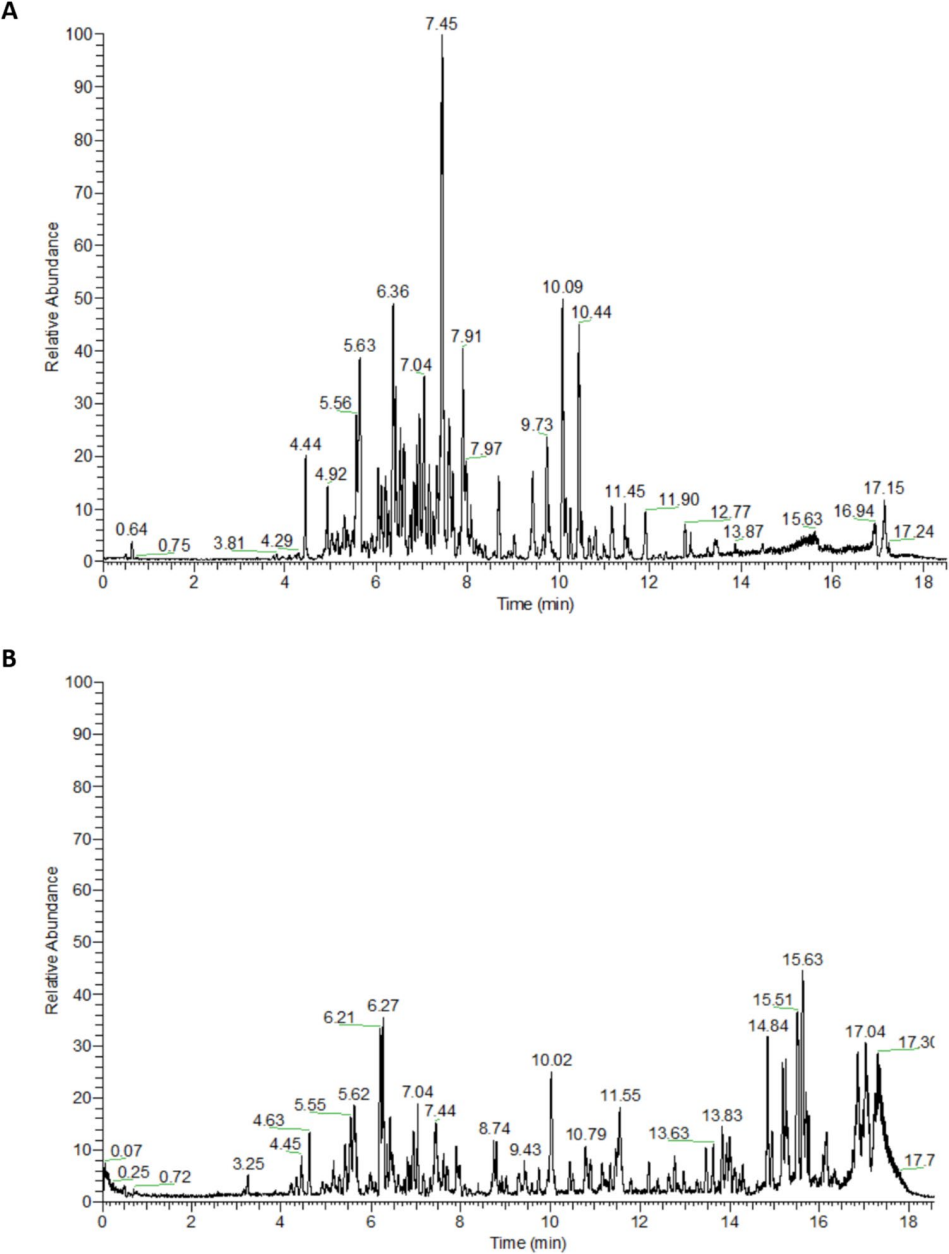
The base peak ion chromatograms in both negative (**A**) and positive (**B**) ionization modes of PG50

The 61 annotated compounds (numbered according to their retention times) belonged to different classes; 32 flavonoids, 13 phenolic acids, 7 coumarins, 5 phenolic glycosides, and 4 dicarboxylic acids (Fig. 4 and Table 5).

**Table 5:** Annotated metabolites of the 50% methanol fraction of *P. graveolens* (PG50) using UPLC-MS/MS in both negative and positive ionization modes

No	Compound name	*R*_*t*_ (min)	Elemental composition	Error (ppm)	*m/z* (+ / −) ppm	MS_n_ ions *m/z* (+ / −) ppm
1	Leonuriside A^d^	3.11	C_14_H_19_O_9_^−^	1.311	331.1037	139, 169
2	Hydroxy methoxy benzoic acid-*O*-hexoside^c^	4.27	C_14_H_17_O_9_^−^	1.491	329.0879	109, 123, 167
3	Dihydroxy coumarin^c^	4.34	C_9_H_5_O_4_^−^	− 0.481	177.0182	97, 133, 149, 159
4	Hydroxy coumarin (Umbelliferone)^e^	4.62	C_9_H_5_O_3_^−^	− 2.013	161.0445	115, 117, 133, 143
5	*O*-Caffeoyl dihydroxy phenyl lactic acid^c^ (rosmarinic acid)	4.72	C_18_H_15_O_8_^−^	0.916	359.0917	135, 161, 179, 197
6	Darendoside A^d^	4.76	C_19_H_27_O_11_^−^	3.53	431.1563	119, 137, 299
7	*O*-Caffeoyl quinic acid^c^	4.88	C_16_H_17_O_9_^−^	3.771	353.0688	179, 191
8	*O*-Caffeoyl quinic acid isomer^c^	4.94	C_16_H_17_O_9_^−^	1.788	353.0873	179, 191
9	*p*-Coumaroyl hexoside^f^	4.99	C_15_H_17_O_8_^−^	1.346	325.0948	101, 119, 163
10	Crosatoside B^d^	5.02	C_20_H_29_O_11_^−^	1.342	445.1722	119, 137, 299
11	Hydroxy cinnamic acid^c^	5.05	C_9_H_7_O_3_^−^	0.259	163.0392	119
12	Phlorisobutyrophenone-hexoside^d^	5.06	C_16_H_21_O_9_^−^	1.311	357.1193	149, 163,177,195
13	Comososide^d^	5.28	C_16_H_23_O_7_^−^	3.914	327.1051	71, 89, 101, 161, 109, 123, 137, 165
14	Coumarin^d^	5.3	C_9_H_7_O_2_^+^	− 2.849	147.0435	103, 119, 129
15	Dihydroxy methoxy coumarin (Fraxetin)^e^	5.3	C_10_H_7_O_5_^−^	− 2.124	207.0284	127, 145, 163, 177, 179, 189
16	Feruloyl tartaric acid^c^	5.35	C_14_H_13_O_9_^−^	2.536	325.0543	149, 193
17	Pimelic acid^d^	5.45	C_7_H_11_O_4_^−^	0.385	159.0656	97, 115, 141
18	Ellagic acid hexoside^b^	5.76	C_20_H_15_O_13_^−^	3.311	463.0519	169, 301
19	Isookanin-*O*-hexoside^c^	5.79	C_21_H_21_O_11_^−^	1.442	449.1089	107, 125, 151, 243, 259, 287
20	*p*-Coumaroyl quinic acid^a^	6.03	C_16_H_17_O_8_^−^	2.031	337.0565	163, 191
21	Myricetin-*O*-hexosyl-deoxyhexoside^f^	6.19	C_27_H_29_O_17_^−^	2.678	625.1417	179, 316, 317
22	Quercetin-*O*-hexosyl-hexoside^b^	6.2	C_27_H_29_O_17_^−^	2.543	625.1393	121, 151, 179, 300, 301
23	Quercetin-*O*-hexoside *O*-hexoside^b^	6.25	C_27_H_29_O_17_^−^	− 2.528	625.1455	121, 151, 179, 300, 301, 463
24	Myricetin-*O*-hexoside^f^	6.35	C_21_H_19_O_13_^−^	0.661	479.0876	61, 179, 316, 317
25	Quercetin *O*-hexoside *O*-pentoside^f^	6.4	C_26_H_27_O_16_^−^	2.62	595.1309	121, 151, 179, 300, 301, 433, 463
26	Ethyl gallate^b^	6.46	C_9_H_9_O_5_^−^	0.52	197.0449	125, 169
27	Hydroxy methoxy benzoic acid^c^	6.54	C_8_H_7_O_4_^−^	0.275	167.0341	108, 123, 152
28	Taxifolin-*O*-hexosyl-deoxyhexoside^c^	6.54	C_27_H_31_O_16_^−^	2.513	611.1518	151, 179, 303
29	Suberic acid^d^	6.64	C_8_H_13_O_4_^−^	0.405	173.0812	111, 129, 155
30	Myricetin-*O*-pentoside^f^	6.72	C_20_H_17_O_12_^−^	2.823	449.0727	151, 179, 242, 271, 287, 316, 317
31	Quercetin-*O*-hexosyl-deoxyhexoside^f^	6.81	C_27_H_29_O_16_^−^	1.919	609.1462	121, 151, 179, 300, 301
32	Myricetin-*O*-deoxyhexoside^f^	6.87	C_21_H_19_O_12_^−^	1.578	463.0888	151, 179, 316, 317
33	Ellagic acid^b^	6.91	C_14_H_5_O_8_^−^	1.126	300.9991	185, 229, 257
34	Quercetin-*O*-hexoside^f^	6.93	C_21_H_19_O_12_^−^	1.212	463.0886	121, 151, 179, 300, 301
35	Quercetin-*O*-hexoside isomer^f^	6.94	C_21_H_19_O_12_^−^	2.136	463.0886	121, 151, 179, 300, 301
36	Kaempferol-*O*-hexoside *O*-pentoside^b^	6.98	C_26_H_27_O_15_^−^	0.887	579.1351	151, 211, 243, 284, 285, 447
37	Kaempferol-*O*-hexosyl-deoxyhexoside^f^	7.35	C_27_H_29_O_15_^−^	2.855	593.1517	151, 229, 243, 257, 285
38	Kaempferol-*O*-hexosyl-deoxyhexoside isomer^b^	7.36	C_27_H_29_O_15_^−^	− 2.742	593.1521	229, 243, 257, 284, 285
39	Quercetin-*O*-pentoside^f^	7.38	C_20_H_17_O_11_^−^	1.402	433.0778	121, 151, 179, 273, 300, 301
40	Isorhamnetin-*O*-hexosyl-deoxyhexoside^b^	7.52	C_28_H_31_O_16_^−^	3.015	623.1625	300, 314, 315
41	Kaempferol-*O*-hexoside^f^	7.6	C_21_H_19_O_11_^−^	1.352	447.0937	151, 199, 243, 284, 285
42	Naringenin-*O*-hexoside^b^	7.78	C_21_H_21_O_10_^−^	0.231	433.1144	107, 119, 151, 271
43	Kaempferol-*O*-pentoside^f^	7.86	C_20_H_17_O_10_-	1.627	417.0833	151, 199, 211, 243, 284, 285
44	Azelaic acid^d^	8.04	C_9_H_15_O_4_^−^	0.485	187.0971	97, 125
45	Isorhamnetin-*O*-hexoside^b^	8.06	C_22_H_21_O_12_^−^	0.118	477.1029	300, 314, 315
46	Myricetin^f^	8.09	C_15_H_9_O_8_^−^	1.296	317.0304	107, 151, 179, 227
47	Methoxy hydroxy cinnamic acid^c^	8.27	C_10_H_9_O_4_^−^	0.525	193.0499	133, 149, 161, 178
48	Isookanin^c^	8.32	C_15_H_11_O_6_^−^	0.836	287.0568	107, 125, 135, 151, 243, 259
49	Kaempferol-*O*-deoxyhexoside^f^	8.42	C_21_H_19_O_10_^−^	2.132	431.0923	151, 199, 211, 243, 284, 285
50	Hydroxy methoxy coumarin^c^	8.62	C_10_H_7_O_4_^−^	2.799	191.0344	129, 145, 147, 161, 163, 173
51	Methoxy coumarin^c^	8.65	C_10_H_7_O_3_^−^	− 1.643	175.0599	131, 145
52	Sebacic acid^d^	9.08	C_10_H_17_O_4_^−^	0.794	201.1129	139, 157, 183
53	Quercetin^f^	9.38	C_15_H_9_O_7_^−^	1.211	301.0355	107, 121, 151, 179, 229, 273
54	Quercetin methyl ether^f^ (Isorhamnetin)	9.92	C_16_H_11_O_7_^−^	1.341	315.0311	135, 243, 254, 255, 271, 272, 300
55	Naringenin^b^	10.35	C_15_H_11_O_5_^−^	1.33	271.0615	93, 107, 119, 151
56	Kaempferol^f^	10.64	C_15_H_9_O_6_^−^	1.316	285.0405	107, 151, 185, 213, 229, 243, 257
57	Kaempferol methyl ether^c^	11.19	C_16_H_11_O_6_^−^	2.693	299.0578	229, 243, 257, 284, 285
58	Luteolin methyl ether^c^	12.57	C_16_H_11_O_6_^−^	2.797	299.0631	151, 175, 199, 241, 284, 285
59	Luteolin dimethyl ether^c^	12.7	C_17_H_13_O_6_^−^	2.042	313.0721	175, 199, 217, 241, 284, 285, 299
60	Hydroxy dimethoxy coumarin (Umckalin)^e^	12.8	C_11_H_9_O_5_^−^	1.578	221.1548	161, 177, 191
61	Methoxyluteolin dimethyl ether^c^	12.83	C_18_H_15_O_7_^−^	1.351	343.0426	199, 241, 267, 270, 285, 313, 328

^a^Metabolite annotation at confidence level 1 is based on a comparison with an internal database of authentic standards analyzed under identical experimental settings

^b,c,d^Metabolite annotation at confidence level 2 is established by referencing dedicated compound databases like HMDB, MassBank, or METLIN, respectively

^e,f^Metabolite annotation at confidence level 2 is achieved by cross-referencing annotated metabolites with existing literature data, either previously isolated compounds or those detected within the genus, respectively

### Molecular Docking Study

**Fig. 5:**
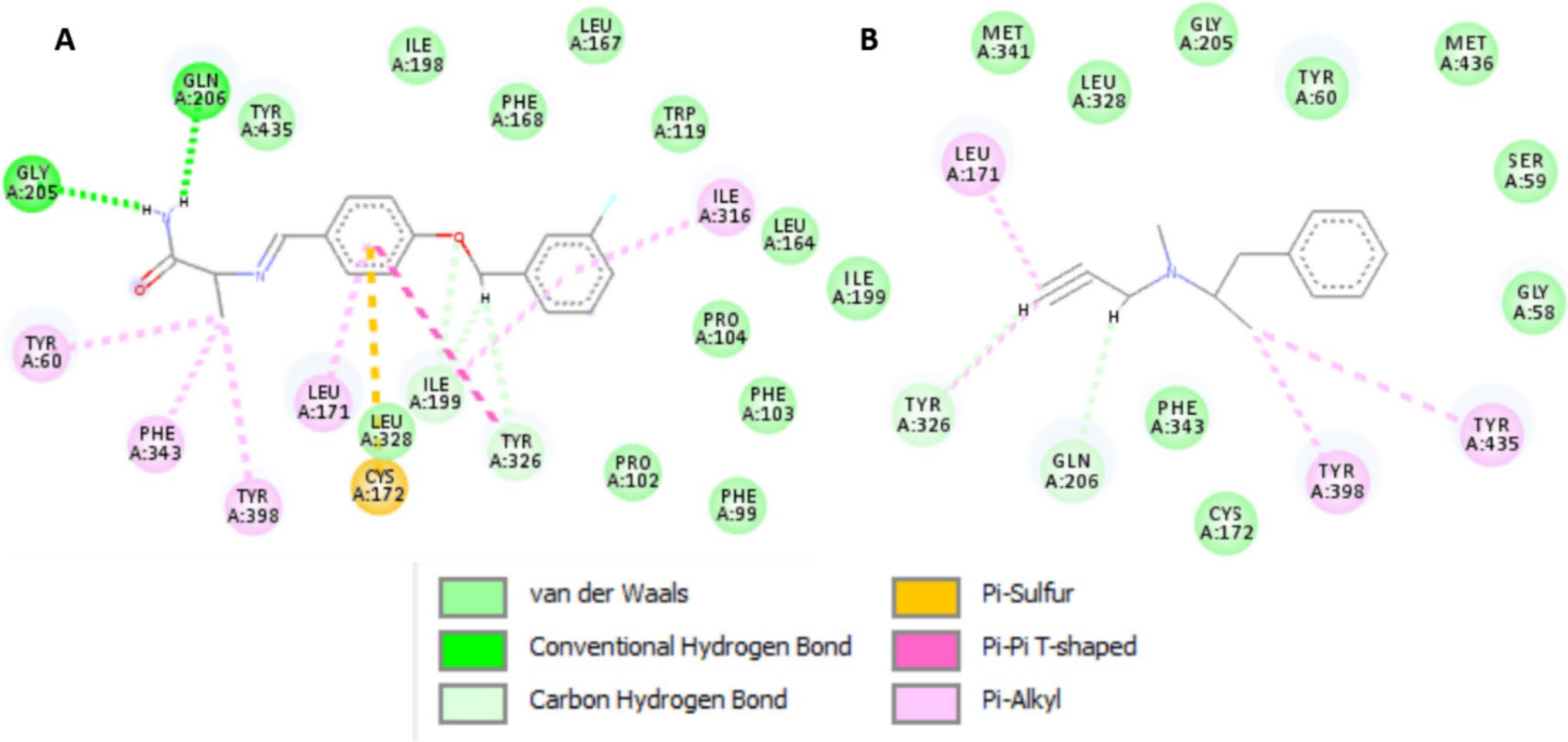
2D-molecular docking simulation of 2 control drugs: safinamide (**A**) and selegiline (**B**) against MAO-B enzyme

**Fig. 6:**
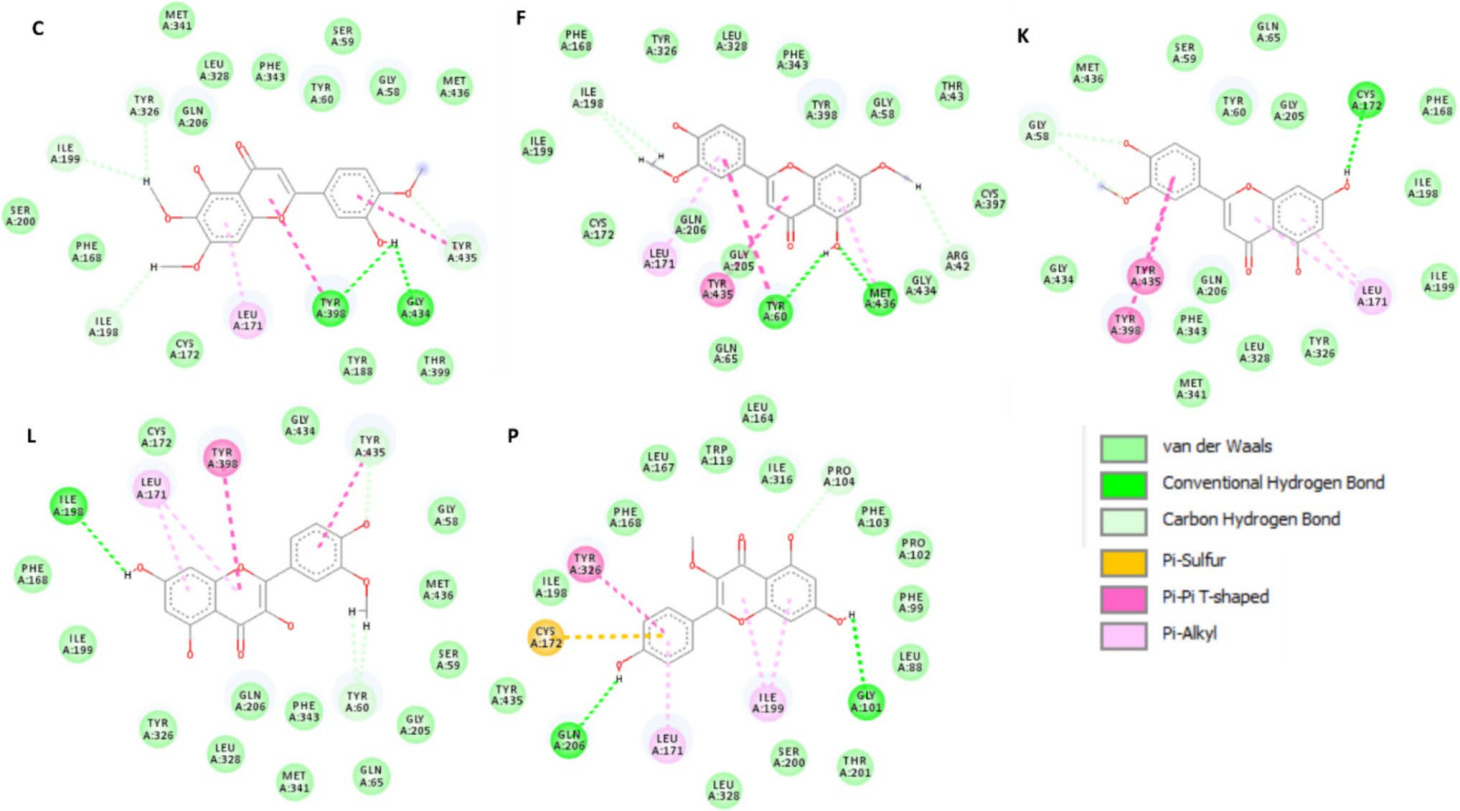
2D-molecular docking simulation of the methoxylated flavonoids having the highest docking scores in PG50 against MAO-B enzyme. Codes are given in Table 1

**Fig. 7:**
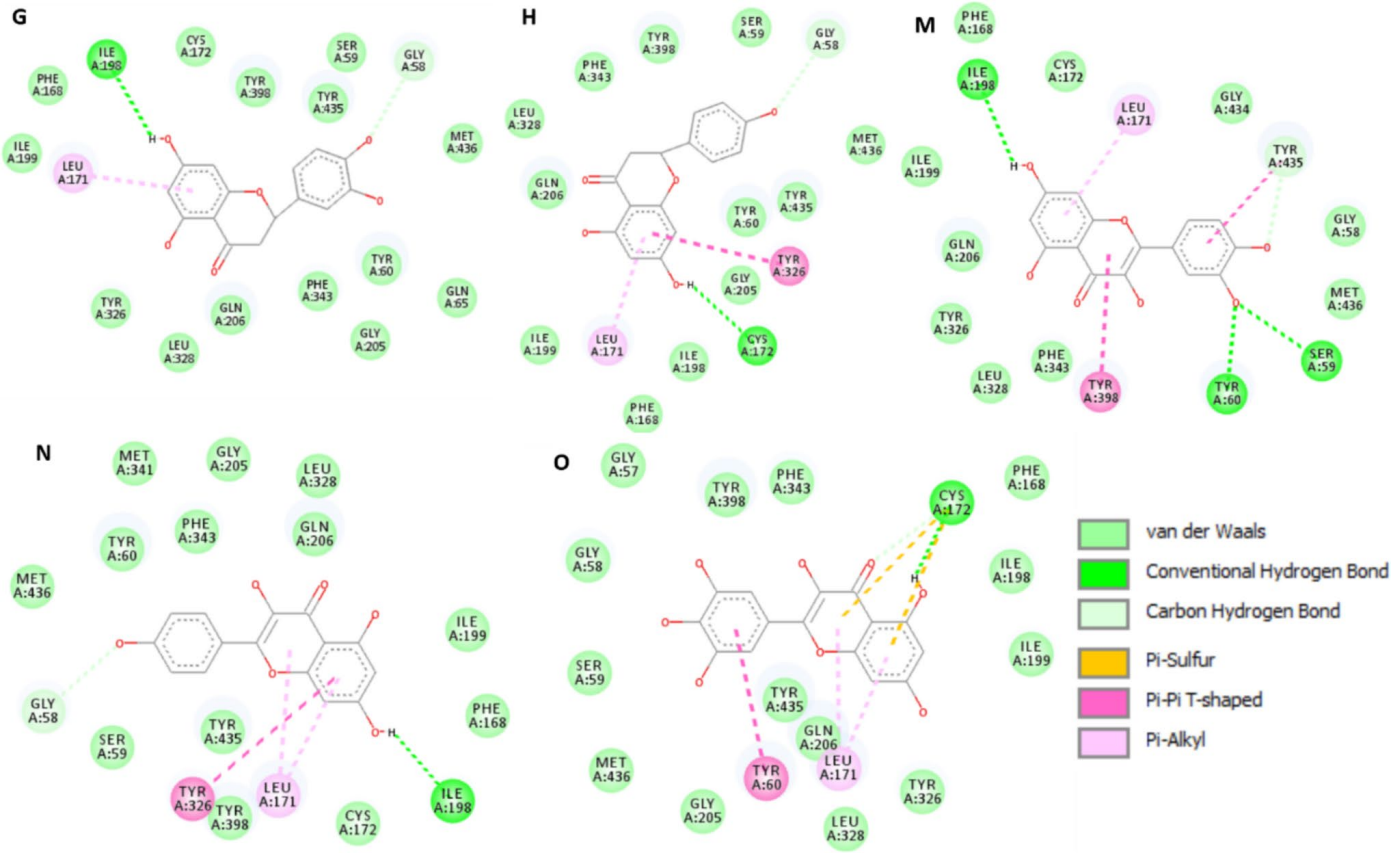
2D-molecular docking simulation of the flavonoid aglycones having the highest docking scores in PG50 against MAO-B enzyme. Codes are given in Table 1

**Fig. 8:**
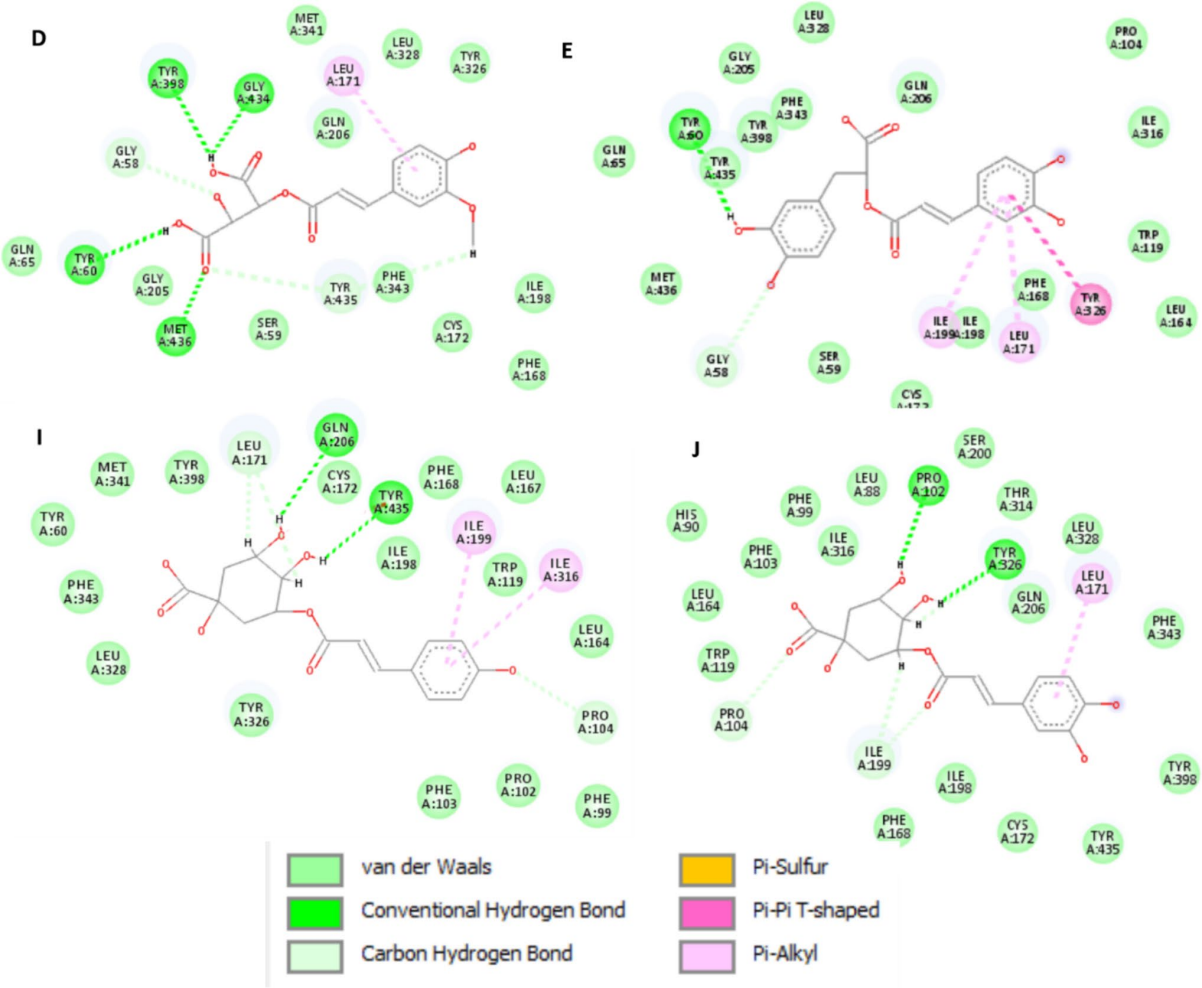
2D-molecular docking simulation of the phenolic acids having the highest docking scores in PG50 against MAO-B enzyme. Codes are given in Table 1

Among the 61 metabolites analyzed, 14 exhibited high affinity to MAO-B enzyme that were comparable to or higher than those of safinamide and selegiline (Table 6). Docking of the two control drugs [safinamide (co-crystallized ligand) and selegiline] in isolated active sites revealed the identification of the important hydrogen bonding and hydrophobic interactions (Fig. 5). Methoxyluteolin dimethyl ether showed the highest docking score of − 8.0625 kcal/mol when compared to safinamide (− 8.2615 kcal/mol), with the same hydrogen bonding (TYR A:326, ILE A:199) and hydrophobic interactions (LEU A:171, TYR A:398). It was well noticed that increasing methoxylation in flavonoids increases the docking score and affinity of the metabolites (Fig. 6): methoxyluteolin dimethyl ether (− 8.0625 kcal/mol), luteolin dimethyl ether (− 7.4038 kcal/mol), and luteolin methyl ether (− 7.2785 kcal/mol). Flavanones such as isookanin (− 7.3767 kcal/mol) and naringenin (− 7.3462 kcal/mol) exhibited higher affinity to MAO-B than flavones such as quercetin (− 6.9617 kcal/mol), kaempferol (− 6.8359 kcal/mol), and myricetin (− 6.7075 kcal/mol) (Fig. 7). Also, cinnamic acids conjugated with organic acids showed high affinity to MAO-B enzyme (Fig. 8). For instance, feruloyl tartaric acid (− 7.7658 kcal/mol) showed hydrogen bonding with MAO-B at TYR A:398, TYR A:60, MET A:436, GLY A:434, GLY A:58, and TYR A:435, as well as hydrophobic interactions at LEU A:171. In addition, *O*-caffeoyl dihydroxy phenyl lactic acid (− 7.5179 kcal/mol) exhibited hydrogen bonding with TYR A:60 and GLY A:58, as well as hydrophobic interactions at LEU A:171, ILE A:199, and TYR A:326.

**Table 6:** In silico docking study results of MAO-B enzyme inhibition (PDB ID: 2V5Z) by various ligands for evaluation of their anti-PD activity

Code	Ligand	Docking score kcal/mol	H-bond interaction	Hydrophobic interaction
A	Safinamide	− 8.2615	TYR A:326, ILE A:199, GLN A:206, GLY A:205	LEU A:171, TYR A:398, PHE A:343, TYR A:60, ILE A:316
B	Selegiline	− 6.2664	TYR A:326, GLN A:206	LEU A:171, TYR A:398, TYR A:435
C	Methoxyluteolin dimethyl ether	− 8.0625	TYR A:326, ILE A:199, ILE A:198, TYR A:435, GLY A: 434, TYR A:398	LEU A:171, TYR A:398, TYR A:435
D	Feruloyl tartaric acid	− 7.7658	TYR A:398, TYR A:60, MET A:436, GLY A:434, GLY A:58, TYR A:435	LEU A:171
E	*O*-Caffeoyl dihydroxy phenyl lactic acid	− 7.5179	TYR A:60, GLY A:58	LEU A:171, ILE A:199, TYR A:326
F	Luteolin dimethyl ether	− 7.4038	ILE A:198, ARG A:42, MET A:436, TYR A:60	LEU A:171, TYR A:435
G	Isookanin	− 7.3767	ILE A:198, GLY A:58	LEU A:171
H	Naringenin	− 7.3462	GLY A:58, CYS A:172	LEU A:171, TYR A:326
I	*p*-Coumaroyl quinic acid	− 7.3602	LEU A:171, GLN A: 206, TYR A: 435	ILE A: 199, ILE A: 316
J	*O*-caffeoyl quinic acid	− 7.3602	TYR A:326, PRO A:102, ILE A:199, PRO A:104	LEU A:171
K	Luteolin methyl ether	− 7.2785	CYS A:172, GLY A: 58	LEU A:171, TYR A:435, TYR A:398
L	Quercetin methyl ether	− 6.9693	ILE A:198, TYR A:435, TYR A:60	LEU A:171, TYR A:398, TYR A:435
M	Quercetin	− 6.9617	ILE A:198, TYR A:60, SER A:59, TYR A:435	LEU A:171, TYR A:398, TYR A: 435
N	Kaempferol	− 6.8359	GLY A:58, ILE A:198	LEU A:171, TYR A:326,
O	Myricetin	− 6.7075	CYS A:172	LEU A:171, TYR A:60
P	Kaempferol methyl ether	− 6.5246	GLN A:206, GLY A:101, PRO A:104	LEU A:171, TYR A:326, ILE A:199

## Discussion

PD primarily affects the central nervous system, especially the basal ganglia, which is essential for regulating voluntary movements [46]. The appearance of PD symptoms such as tremors and bradykinesia arise from a complex interaction involving the degeneration of dopaminergic neurons in the SN, the aggregation of α-Syn, neuroinflammation, and mitochondrial dysfunction [47].

Although the exact cause of PD remains unknown, both genetic and environmental factors are believed to play a role [48]. Some genetic mutations have been identified as risk factors for developing PD, but they account for a small percentage of cases. Environmental factors, including exposure to certain toxins and pesticides such as rotenone, have also been implicated [49].

In this study, the bioactive fraction PG50 exhibited the highest inhibitory activity against the MAO-B enzyme *in vitro* among all the fractions, and its neuroprotective effect was subsequently evaluated in vivo using a rotenone-induced PD rat model.

The MAO-B assay and the rotenone-induced PD rat model are connected through their role in oxidative stress and dopaminergic neurodegeneration, which are central to PD pathology [50, 51]. MAO-B is an enzyme that metabolizes DA in the brain, producing hydrogen peroxide as a byproduct. Elevated MAO-B activity contributes to increased oxidative stress, which exacerbates the loss of dopaminergic neurons in the SN, a hallmark of PD [52]. Rotenone, a mitochondrial complex I inhibitor, induces oxidative stress and mimics PD-like symptoms, including motor deficits and dopaminergic neurodegeneration in animal models [53].

The *in vitro* MAO-B inhibition assay demonstrated that the PG50 effectively inhibited MAO-B activity, indicating its potential to reduce oxidative stress. This was further supported by the in vivo rotenone-induced PD rat model, where PG50 alleviated motor deficits, restored neurotransmitter levels, reduced oxidative damage, and mitigated neuroinflammation. The reduction in α-Syn accumulation and the halting of histopathological changes in the SN also highlight its neuroprotective effects. Thus, the MAO-B inhibition observed *in vitro* directly correlates with the reduction of oxidative stress and neurodegeneration seen in vivo, establishing PG50 as a promising therapeutic candidate for managing PD.

Motor coordination and balance in the animals were assessed using the wire-hanging test. The improvement in motor impairments observed in the PD rats treated with PG50, as indicated by the increased duration of balance on the rod, suggests that PG50 may have a positive effect on motor coordination and balance control. This effect may be due to the high phenolic and flavonoid content observed in our UPLC-MS/MS results, where a review by Khazdair et al. (2021) signifies a promising aspect of phenolic compounds in mitigating the behavior deficits associated with PD in various experimental models [54].

The α-Syn lesions replicate and spread between the interconnected brain regions where it is deposited in fibrillary structures called Lewy bodies (LB) [4]. Sivanesam and Andersen (2016) suggest that soluble β-oligomers, formed prior to the development of mature fibrils, are likely responsible for cellular toxicity, possibly by creating membrane pores [55]. Thus, targeting protein aggregation and oligomerization emerges as a promising strategy in combatting neurodegeneration. Our findings indicated a reduction in α-Syn levels after treatment with PG50, which may be due to its high content of flavonoids and phenolic acids such as myricetin and rosmarinic acid. For instance, Ono and Yamada (2006) utilized fluorescence spectroscopy with thioflavin S and electron microscopy to study the effects of 13 antioxidants on α-Syn fibril formation and destabilization and found that rosmarinic acid and myricetin exhibit anti-fibrillogenic and fibril-destabilizing properties, potentially preventing the onset of α-Syn aggregation by inhibiting fibril deposition in the brain [56].

Rotenone is also known to induce oxidative stress, reduce DA levels, and cause neurotoxicity in SN of rats [57]. This aligns with our findings, which showed a reduction in DA levels in rotenone-induced rats. In line with the current findings, levels of NE and 5-HT decreased in the CNS, and has an impact on cerebral function in people with PD [58]. After the treatment of PD rats with PG50, we noticed an improvement in the levels of DA, 5-HT, and NE which may be due to the presence of coumarins such as 7-hydroxycoumarin, esculetin, and umbelliferone that emerged as the most extensively studied compounds known for their effect on normalizing the level of these neurotransmitters [59]. Also, flavonoids such as quercetin (50 mg/kg) were proven to increase the DA level in 6-OHDA-induced PD in rats [60].

Studies have shown that alterations in antioxidant defenses support the idea that oxidative stress plays a significant role in the development of PD [61]. The most notable disruption in the antioxidant defense system includes a decrease in glutathione (GSH) levels, reduced activity of superoxide dismutase (SOD), and increased lipid peroxidation (indicated by increased MDA). These conditions lead to excessive oxidative stress and neuronal death. Our findings align with this, as we observed reduced GSH level and SOD activity along with increased MDA levels in rats with PD induced by rotenone. The normalization of these markers was achieved following the treatment with PG50 which may be due to its high content of phenolic compounds. A review by Sun et al. (2023) highlighted the neuroprotective effects of natural phenolics, emphasizing their role in modulating oxidative stress markers like GSH, SOD, and MDA [62]. These results are in line with several studies that showed the effect of various *P. graveolens* extracts on oxidative stress, particularly their effects on GSH, SOD, and MDA levels, which can be attributed to their phenolic content [63, 64].

Succinate dehydrogenase (SDH) is an enzyme involved in the mitochondria’s oxidative phosphorylation system, linking the Krebs cycle to the electron transport chain. Dysfunction or inhibition of SDH can disrupt mitochondria and impair ATP levels. This is consistent with the observed decrease in SDH levels in rats with rotenone-induced PD, suggesting that balancing SDH may play a significant role in neuroprotection. Also, lactate serves as an alternative source of energy for the brain under certain conditions. It can be oxidized in the mitochondrial TCA cycle, generating ATP. The brain’s ability to produce and utilize lactate is regulated by the activity ratios of lactate dehydrogenase (LDH) isoenzymes. An increased LDH-A/LDH-B gene expression ratio indicates high brain lactate levels, which may be indicative of aging. In the case of rotenone-induced PD rats, the decreased LDH enzyme levels in brain tissue can be attributed to the excessive conversion of pyruvate to lactate. PG50 enhanced the levels of both SDH and LDH, likely due to its high content of phenolics, which serve as potent antioxidants that protect cells from oxidative damage. These phenolics may contribute to increased SDH and LDH activity by promoting better mitochondrial health and improving aerobic conditions [65].

In terms of inflammatory mediators, our study observed elevated levels of IL-6 in PD, consistent with previous studies [66]. IL-6 can promote leukocyte adhesion and migration, disrupt the blood–brain barrier, and activate glial cells. This cascade of events can lead to the overproduction of reactive oxygen species, apoptosis, and cell death. Glial activation is implicated in neurodegenerative diseases, and inflammation can exacerbate hypoxia by damaging mitochondria and endothelial cells, which impairs blood flow regulation. Other studies have also reported elevated levels of IL-6 and TNF-α in PD patients, which leads to degeneration and loss of dopaminergic neurons. The inflammatory markers IL-6 and TNF-α were potentially improved by PG50, supported by a study conducted by Ghanizadeh et al. (2015), which demonstrated the potential anti-inflammatory effects of the methanol extract of *P. graveolens* [21].

While the *in vitro* inhibitory effects of PG50 on MAO-B were significant, it is essential to highlight how this activity may translate into potential therapeutic benefits. Discussing the chemical diversity of PG50 can further strengthen the rationale for its selection. Data showed the weights of the total extract and its fractions: the total extract weighed 80 g, with the three fractions being a 100% water fraction (12 g), a 50% methanol fraction (50 g), and a 100% methanol fraction (14 g). The 50% methanol fraction (PG50) has the greatest weight, suggesting that it may comprise most of the compounds from the total extract, potentially contributing to synergistic effects. Additionally, metabolite profiling of PG50 using UPLC-MS/MS analysis identified a wide range of metabolites from various phytochemical classes such as flavonoids, phenolic acids, coumarins, phenolic glycosides, and dicarboxylic acids which are predominantly extracted using the 50% methanol–water solvent. Most of these compounds from the different classes have demonstrated potential as neuroprotective agents in various models of PD [67]. For instance, several studies proved that methoxylated flavonoids has the ability to be potent antioxidants and neuroprotective agents against neurotoxin-induced neurotoxicity. They significantly reversed memory and motor deficits, and normalized biochemical anomalies [68, 69]. Also, flavonols such as quercetin is emphasized for its neuroprotective effects and potential health benefits in various neurodegenerative diseases [70]. Furthermore, a clinical trial included 49,281 men from the Health Professionals Follow-up Study and 80,336 women from the Nurses’ Health Study, focusing on five major sources of flavonoid-rich foods: tea, berry fruits, apples, red wine, and orange/orange juice. Results showed that higher intakes of flavonoids were significantly linked to a reduced risk of PD [71]. Moreover, researchers previously concentrated on the antioxidant and anti-inflammatory properties of phenolic acids. However, interest has recently surged in the neuroprotective effects of phenolic acids on neurons and glial cells, resulting in a significant increase in studies addressing their role in neuroprotection [72].

Further assessment of these metabolites was performed in an in silico study focusing on MAO-B enzyme inhibition. A key objective is to correlate the observed anti-PD activity of PG50 with its active constituents and to explore potential new drugs derived from natural sources for treating PD. Safinamide (co-crystallized ligand) and selegiline, known as MAO-B inhibitors, were used as reference drugs [73]. Redocking of the co-crystalized ligand in the isolated active sites revealed the importance of validating the study and defining the accurate binding poses and interactions for understanding ligand-receptor interactions and binding affinities. It is well noticed that most of the 14 metabolites exhibited the hydrophobic interaction with LEU A:171 which is situated near the substrate-binding pocket of MAO-B and plays a pivotal role in stabilizing interactions with metabolites. The hydrophobic nature of leucine allows it to engage effectively with other hydrophobic residues and the aromatic components of substrates, which enhances the binding affinity [74]. Also, Hubalek et al. (2005) explored Ile199’s impact on MAO-B selectivity, highlighting the formation of the enzyme’s “aromatic cage” by FAD, Tyr398, and Tyr435 [75]. Recent investigations focus on developing selective MAO-B inhibitors due to the risk of hypertensive crises from MAO-A inhibition [76]. Docking studies with potent ligands revealed strong hydrophobic interactions with Ile199, Tyr398, and Tyr435, alongside a hydrogen bond with Tyr326, underscoring Tyr326’s significance in MAO selectivity [77].

Our research findings suggest that methoxylation for flavonoids can enhance their affinity to MAO-B, as indicated by increased docking scores; methoxyluteolin dimethyl ether > luteolin dimethyl ether > luteolin methyl ether. This finding aligns with the study conducted by Chaurasiya et al. (2020), which showed that methoxylation patterns played a critical role in enhancing selectivity and potency by increasing hydrophobic interactions, and optimizing binding to active sites of MAO-B. The tetra-methoxylation in the compound “5,7-dihydroxy-2,3,4,5-tetramethoxyflavone” facilitated strong hydrophobic and hydrogen-bond interactions with key MAO-B residues, such as Tyr435, Cys172, and Gln206, including water-mediated hydrogen bonds. Experimental data suggested that methoxylated flavonoids exhibit comparable or superior inhibitory activity to standard inhibitors such as deprenyl [78]. Moreover, flavanones showed a higher affinity to MAO-B compared to flavonols, which also aligns with the work of Pannu et al. (2021) who found that substituting the C3 position with a longer chain reduces the capacity to inhibit MAO enzymes [79]. Additionally, hydroxylation, particularly in the ring B, improves the inhibition of MAO-B due to desired hydrogen bonding (quercetin > kaempferol). Conversely, tri-substitution in the ring B of flavonoids (such as myricetin) reduces hydrogen bonding essential for MAO-B inhibition, likely due to steric hindrance, altered electronic properties, conformational changes, and impaired binding site accessibility [80].

## Conclusion

In summary, this research highlights the neuroprotective properties of the bioactive fraction of *P. graveolens* (PG50) in a rat model of rotenone-induced Parkinson’s disease. The treatment enhanced motor and behavioral functions, normalized neurotransmitter levels, reduced α-Syn aggregation, restored oxidative balance, decreased inflammation markers, and enhanced mitochondrial enzyme functions. UPLC-MS/MS revealed the annotation of 61 metabolites across various chemical classes including flavonoids, phenolic acids, and coumarins. The in silico study revealed that methoxylated flavonoids, cinnamic acids conjugated with organic acids, and flavonoid aglycones have high affinity to MAO-B enzyme, with methoxyluteolin dimethyl ether as a promising lead compound for MAO-B inhibition. These findings were supported by docking scores, and the study aimed to establish a correlation between the anti-PD activity of PG50 and its active constituents while exploring potential natural-based drugs for PD treatment.

## Data Availability

The manuscript contains all the data that support the findings.
